# The Prognostic Role of the Surgical Margins in Squamous Vulvar Cancer: A Retrospective Australian Study

**DOI:** 10.3390/cancers12113375

**Published:** 2020-11-14

**Authors:** Ellen L. Barlow, Michael Jackson, Neville F. Hacker

**Affiliations:** 1 Gynaecological Cancer Centre, Royal Hospital for Women, Sydney 2031, Australia; n.hacker@unsw.edu.au; 2 Radiation Oncology Department, Prince of Wales Hospital, Sydney 2031, Australia; Michael.Jackson@health.nsw.gov.au; 3 Prince of Wales Clinical School, University of New South Wales, Sydney 2052, Australia; 4 School of Women’s & Children’s Health, University of New South Wales, Sydney 2052, Australia

**Keywords:** squamous vulvar carcinoma, vulvar conservation, resection margin, local recurrence, primary site recurrence, remote site recurrence

## Abstract

**Simple Summary:**

Squamous cell carcinoma of the vulva is a rare disease, but cure rates are good if managed appropriately. The need for radical vulvectomy was initially challenged about 40 years ago for lesions 1−2 cm diameter. Since then, there has been progressive acceptance of radical local excision for most unifocal squamous vulvar cancers. Originally, a surgical margin of 3 cm around the primary cancer was considered appropriate. Subsequently, a 1 cm margin was generally accepted, but this has become the subject of recent debate. The aims of this study were to determine survival following conservative vulvar resection, and to determine the clinicopathological predictors associated with vulvar recurrence, focusing on the surgical margin. In multivariable analysis, primary site recurrences were increased in patients with margins < 8 mm, and all vulvar and primary site recurrences in patients with margins < 5 mm. Treatment of close or positive margins decreased the risk of recurrence.

**Abstract:**

For the last 30 years at the Royal Hospital for Women, unifocal vulvar squamous cancers have been treated by radical local excision, aiming to achieve a histopathological margin of ≥8 mm, equating to a surgical margin of 1 cm. The need for a margin of this width has recently been challenged. We aimed to determine the long-term outcome following this conservative approach, and the relationship between vulvar recurrences and surgical margins. Data were obtained retrospectively on 345 patients treated primarily with surgery for squamous vulvar cancer between 1987 and 2017. Median follow-up was 93 months. Five-year disease-specific survival was 86%. Of 78 vulvar recurrences, 33 (42.3%) were at the primary site and 45 (57.7%) at a remote site. In multivariable analysis, a margin < 5 mm showed a higher risk of all vulvar (Hazard ratio (HR), 2.29; CI, 1.12−4.70), and primary site recurrences (subdistribution hazard ratio (SHR), 15.20; CI, 5.21−44.26), while those with a margin of 5 to <8 mm had a higher risk of a primary site recurrence (SHR, 8.92; CI, 3.26−24.43), and a lower risk of remote site recurrence. Excision margins < 8 mm treated by re-excision or radiation therapy had a significantly decreased risk of recurrence. Guidelines should continue to recommend a surgical margin of 1 cm.

## 1. Introduction

Squamous cell carcinoma of the vulva (VSCC) is a rare disease, but cure rates are good if the primary tumour and groin lymph nodes are managed appropriately [1]. In the mid-20th century, radical vulvectomy was the standard of care for the primary tumour, although it caused serious psychosexual morbidity [2]. The main argument against performing a more conservative vulvar operation was that multicentricity occurred in 20−30% of cases, and more conservative procedures would result in more vulvar recurrences [3]. This concern has been explained by the concept of field cancerization [4].

The need for radical vulvectomy in all patients was initially challenged about 40 years ago for lesions up to 1 cm [5] and 2 cm [6] in diameter. Since that time, there has been a slow acceptance of radical local excision for most unifocal squamous vulvar cancers [7,8], although the change in management has never been subjected to a prospective, randomized trial. 

The width of the surgical margin has remained controversial. In the original papers, a 3 cm margin of normal skin on all sides of the primary cancer was considered appropriate [5,6,7]. In a 1990 study of 135 patients, 81.5% of whom had early stage disease, Heaps et al. reported that an 8 mm histological tumour-free surgical margin resulted in a high rate of local control, whereas a margin of <8 mm was associated with a 50% chance of local recurrence [9]. With tissue shrinkage after formalin fixation, an 8 mm histological margin equated to a surgical margin of 1 cm. Subsequently, a 1 cm disease-free margin was generally accepted as being appropriate, although in 2002, deHullu et al. recommended that the margin should be increased to 2 cm [10].

An important new concept regarding local vulvar relapse was introduced by Rouzier and colleagues in 2002 [11]. They proposed that vulvar relapses should be divided into (i) primary site and (ii) remote site recurrences. They defined primary site recurrences as those involving the skin within 2 cm of the vulvectomy scar, and remote site recurrences as those occurring further away. On multivariable analysis, primary site recurrences were significantly related to surgical margins less than 1 cm and had a mean time to recurrence of 13 months. Remote site recurrences often arose from epithelial disorders, such as lichen sclerosis, and the mean disease-free interval was 33 months. They considered remote site recurrences to be new primary cancers.

Recently, the relevance of 1cm surgical margins has been questioned [12,13,14,15,16,17,18]. We have had a consistent policy of vulvar conservation for unifocal vulvar cancer at the Royal Hospital for Women in Sydney since 1987, so we decided to review our experience. Our primary aims were to determine the relationship between local vulvar recurrence and the extent of the histopathological surgical margin, and to explore patterns of local recurrence. A secondary aim was to determine the long-term survival of patients treated with a conservative approach to the primary lesion. 

## 2. Results

### 2.1. Primary Treatment and Staging

The study population included 345 patients treated primarily with surgery for VSCC. Table 1 shows a summary of the clinicopathological and treatment characteristics for these patients. The patients were followed for a median of 93 months (range 1−367 months). All FIGO stages were represented, except FIGO stage IVA^(i)^. 

Of the 63 patients who did not have a groin lymphadenectomy, 31 had Stage IA disease and no lymphadenectomy was offered. A further 31 patients had stage IB disease. Twenty-one of these patients had tumours 15 mm or less in diameter, their groin nodes were followed with ultrasound for 12 months post-operatively on a research protocol, and all remained negative. Of the remaining 10 patients, lymphadenectomy was contraindicated in six because of poor performance status and four refused any lymphadenectomy. None of these 31 patients developed a groin recurrence with a minimum follow-up of 30 months and they were regarded as node negative for analysis. One patient with stage II disease refused a lymphadenectomy. She was treated with radiotherapy to the groin and pelvis and her nodal status was recorded as unknown. 

### 2.2. Survival

At the completion of the study, of the 345 patients treated primarily with surgery with curative intent, 209 patients (60.6%) were alive without evidence of disease, and 136 patients (39.4%) had died. Of the 136 deaths, 55 patients (15.9%) died of disease, two died from peri-operative complications (0.6%) and one died of sepsis during adjuvant radiotherapy (0.3%). The remaining 78 patients died of unrelated causes (22.6%). 

**Figure 1:**
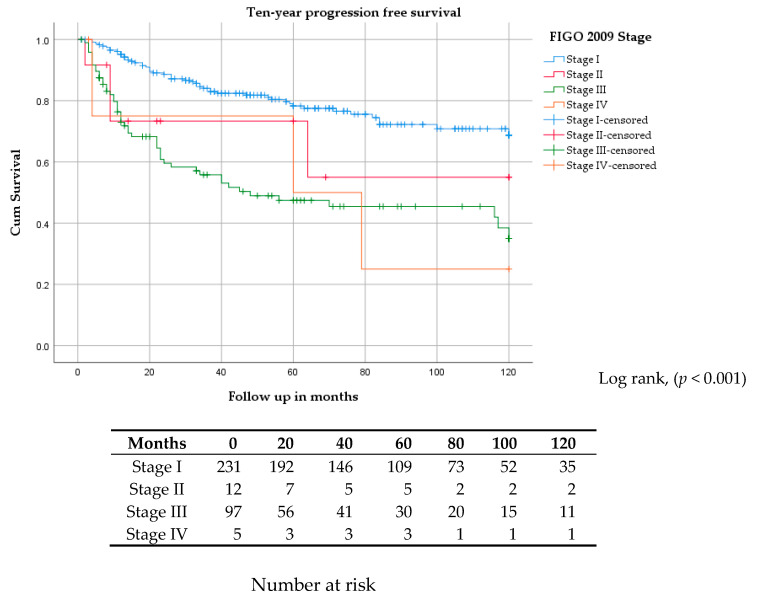
Kaplan–Meier curve for ten-year progression-free survival stratified by FIGO stage.

**Figure 2:**
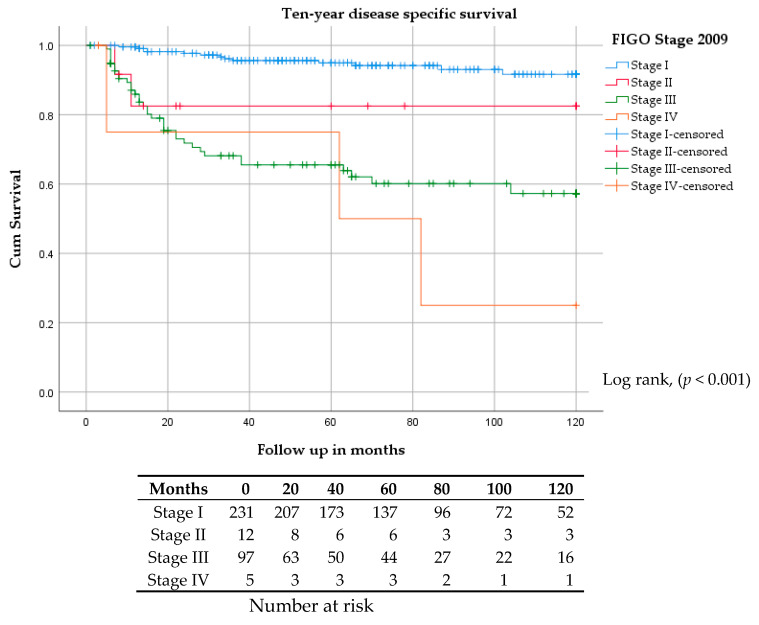
Kaplan–Meier curve for ten-year disease-specific survival stratified by FIGO stage.

Kaplan–Meier estimates for five and ten-year progression-free survivals (PFS) were 78% and 70% for stage I, 75% and 55% for stage II, 47% and 35% for stage III, and 50% and 26% for stage IV, respectively (Figure 1). Five and ten-year disease-specific survivals (DSS) were 95% and 92% for stage I, 82% and 82% for stage II, 66% and 58% for stage III, and 75% and 25% for stage IV, respectively (Figure 2). 

The five and ten-year DSS for all 345 patients was 86% and 80%, respectively (Figure S1), and for the 102 patients with positive lymph nodes it was 66% and 55%, respectively (Figure S2). The five and ten-year DSS was 94% and 80%, for the 69 patients with an isolated vulvar recurrence, and 8% and 4% for the 42 patients with a regional or distant recurrence (Figure S3). The five and ten-year overall survival (OS) for all 345 patients was 73.6% and 64.3%, respectively (Figure S4).

### 2.3. Surgical Margins

**Figure 3:**
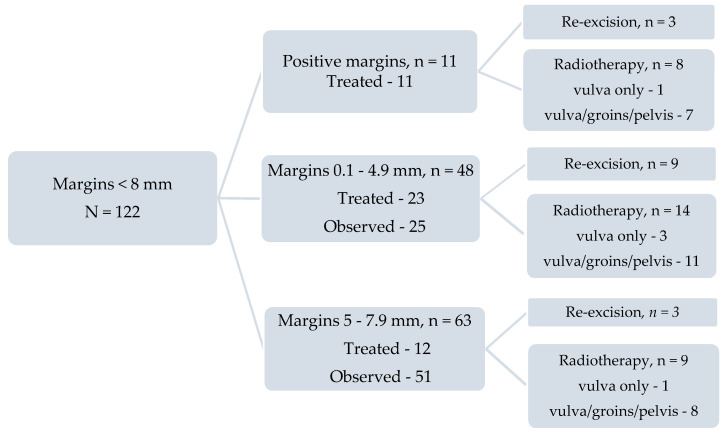
The relationship between surgical margins < 8 mm and subsequent management.

To determine the relationship between peripheral margin distance and vulvar recurrence, patients with less than six months follow-up, none of whom had a vulvar recurrence, were excluded (*n* = 4). Of the remaining 341 patients, 219 (64.2%) had a histopathological margin distance ≥8 mm, 111 (32.5%) had a margin between 0.1 mm and 8 mm (close margins) and 11 (3.2%) had positive margins. Of the 111 patients with close margins, 48 patients had a margin between 0.1 mm and <4.9 mm, and 63 between 5 mm and 7.9 mm (Table S1). Of the 122 patients with close or positive margins, 46 (37.7%) were treated by re-excision or adjuvant radiation therapy and 76 (62.3%) were observed. The relationship between surgical margins and subsequent management is shown in Figure 3 and Table S1. All ‘surgical margins’ or ‘margins’ referred to in this paper are the histopathological surgical margin distance as measured by the pathologist and recorded in the histopathology report. 

### 2.4. Recurrences

There were 111 recurrences (32.2%). Of these, 78 (70.3%) were on the vulva (seven concurrent with a groin recurrence, one concurrent with a vaginal recurrence, and one concurrent with a distant recurrence). Thirty-four of the 78 vulvar recurrences (43.6%) developed two or more vulvar recurrences. Of the remaining 33 recurrences, 14 were distant, 11 isolated groin, two isolated vagina, one isolated skin bridge, three concurrent groin and distant, one concurrent groin and vagina, and one concurrent groin and skin. Among those with a groin or distant recurrence, the median interval to a groin recurrence was 8 months, (range 3–22 months) and the median interval to a distant recurrence was 11 months, (range 2–79 months). Of the 78 vulvar recurrences, 33 (42.3%) were at the primary site and 45 (57.7%) were at a remote site. The median interval from initial treatment to a primary site recurrence was 20 months compared to 39 months for a remote site recurrence. For both vulvar sites, the earliest first recurrence occurred at two and seven months, respectively. Two primary and 14 remote site recurrences developed more than five years after the initial treatment. 

### 2.5. Relationship between Vulvar Recurrence and Various Histopathological and Other Factors

#### 2.5.1. Surgical Margins

Vulvar recurrences occurred in 51 of 219 patients (23.3%) with pathological margins ≥8 mm, compared to 27 of 122 patients (22.1%) with pathological margins < 8 mm (*p* = 0.65) (Figure S5). However, vulvar recurrences occurred in 23 of 76 patients with untreated margins < 8 mm (30.2%) compared to four of 46 patients with treated close or positive margins (8.7%) (*p* = 0.005) (Table S1). 

For patients with margins 0.1–4.9 mm, treatment with either radiotherapy or re-excision decreased the rate of primary site recurrence from 40 to 4.3% (*p* = 0.003). For each individual modality, re-excision decreased the recurrence rate from 40 to 11.1% (*p* = 0.09), and radiation from 40% to 0% (*p* = 0.009) (Table S1). A per mm breakdown of the incidence of recurrence in untreated patients whose margins were <5 mm is shown in supplementary Table S2. Treatment for patients with margins 5–7.9 mm was less beneficial, although treatment with either modality was associated with a decreased rate of recurrence from 25.5 to 11.1% (*p* = 0.165) (Table S1). 

**Figure 4:**
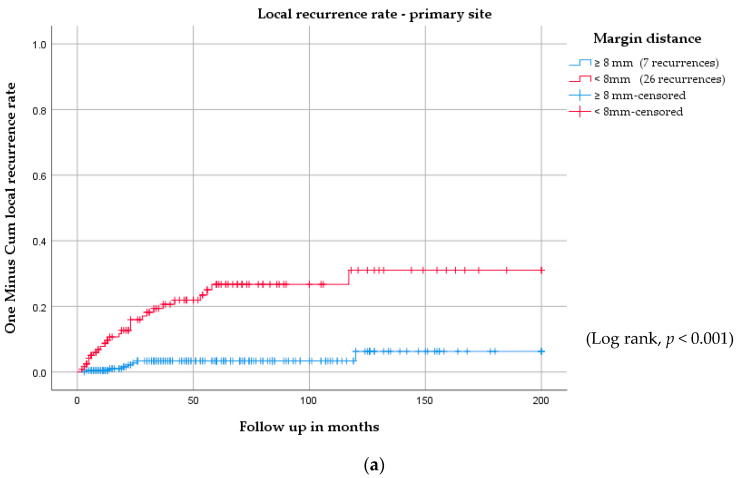

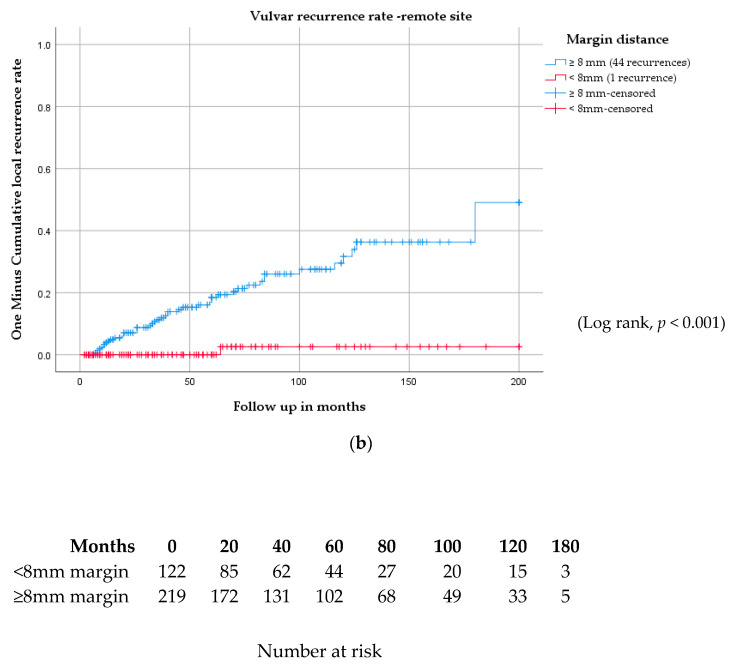
Vulvar recurrence rate for (**a**) the primary site and (**b**) the remote site stratified by surgical margins of <8 mm and ≥8 mm.

There were 27 vulvar recurrences in patients with margins < 8 mm, of which 26 were at the primary site, and one was at a remote site (*p* < 0.001). Of the 51 recurrences in patients with margins ≥ 8 mm, 44 were at a remote site and seven were at the primary site (*p* < 0.001) (Figure 4, Table S1). 

For univariable (Table 2) and multivariable (Table 3) Cox and Fine–Gray regression analyses, patients were categorised using the margin cut offs <5 mm, and 5 to <8 mm, compared to ≥8 mm. We identified significant differences in local recurrence rates related to pathological margin distance and the location of a recurrence on the vulva (primary or remote site). In univariable analysis, although we found no increased risk for all vulvar recurrences in patients with margins < 8 mm, primary site vulvar recurrences occurred with increased risk in both close margin groups; <5 mm (Sub-distribution hazard ratio [SHR], 8.82; CI, 3.54−21.99), and 5 mm to <8 mm (SHR, 7.59; CI, 3.04−18.95). This result was consistent in multivariable analysis, where both margin groups < 8mm maintained a significantly increased risk for primary site recurrence. In addition, in the pathological margin group < 5 mm, there was evidence of an increased risk for all vulvar recurrences (Hazard ratio (HR), 2.29; CI, 1.12–4.70). There was a decreased risk for remote vulvar recurrence in patients with margins 5mm to <8 mm in the univariable (SHR, 0.07; CI, 0.01−0.54) and multivariable analysis (SHR, 0.08; CI, 0.01−0.71). There were no remote vulvar recurrences in the <5 mm margin group.

For patients with positive or close margins < 8 mm, those treated by surgical re-excision or radiation therapy had a significantly lower risk of all vulvar recurrences in univariable analysis (HR, 0.34; CI, 0.01−0.54). However, in multivariable analysis, treatment was strongly associated with a decreased risk for all vulvar recurrence (HR, 0.15; CI, 0.05−0.44), and for a primary site recurrence (SHR, 0.14; 0.05−0.41). 

#### 2.5.2. Epithelial Abnormality

In univariable analysis, the presence of an epithelial abnormality (compared to no epithelial abnormality) was not associated with a significantly increased risk for a vulvar recurrence at any site. For patients with differentiated vulvar intraepithelial neoplasia (dVIN) in the specimen, we observed no significantly increased rate of any vulvar recurrence in univariable or multivariable analyses. In multivariable analysis, dVIN at the excision margin showed a significantly increased risk for recurrence at the primary vulvar site (SHR, 5.35; CI, 1.05−27.34). In univariable analysis, the presence of usual-type vulvar intraepithelial neoplasia (uVIN) in the specimen was associated with a lower rate of all vulvar recurrences (HR, 0.43; CI, 0.20−0.96), but not primary or remote vulvar site recurrences compared to no epithelial abnormality. However, this was not significant in multivariable analysis (*p* = 0.06).

#### 2.5.3. Positive Nodes

In both the univariable and multivariable analysis, patients with one or more positive lymph nodes had an increased risk of all vulvar recurrences, and primary site vulvar recurrence.

#### 2.5.4. Lymphovascular and Perineural Space Invasion

In univariable analysis, lymphovascular space invasion (LVSI) and perineural invasion (PNI) were associated with an increased risk of recurrence, but only at the primary vulvar site; LVSI, (SHR, 2.67; CI, 1.21−5.91) and PNI, (SHR, 3.12; CI, 1.08−9.06). This was not confirmed in the multivariable analysis.

#### 2.5.5. Other Factors

In univariable analysis, current smokers had a significantly lower risk of vulvar recurrence at all vulvar sites, and this remained in multivariable analysis (HR, 0.42; 0.20−0.87). The evidence of lower risk was not as strong when primary or remote site recurrences were considered separately. Tumour diameter, grade of differentiation, and depth of invasion had no significant influence on local recurrence rates in either univariable or multivariable analysis. Multivariable analysis also identified a decreased risk for all vulvar recurrences in women older than 65 years (HR, 0.58; CI, 0.33–1.00). 

## 3. Discussion

This study demonstrates that a conservative surgical approach to vulvar cancer is associated with excellent rates of survival. The old standard treatment was radical vulvectomy and bilateral groin dissection, with or without pelvic node dissection in all patients. A report of 96 patients with T1 and T2 lesions from the United States in 2007 reported radical vulvectomy in 49.2% of cases and bilateral groin dissection in 51% [19]. A Surveillance, Epidemiology and End Results (SEER) study of 141 patients with FIGO stages I/II disease treated in 1999 reported that 47% of patients were treated with a radical vulvectomy [20]. More recently, a 2020 study, which included a cohort of 1535 patients treated for all stages of squamous vulvar cancer between 2001 and 2005 across 100 European centres reported radical vulvectomy in 76.5% and bilateral groin dissection in 45.2% of patients [21]. 

By contrast, only 20% of patients with all stages of disease in this study had radical vulvectomy, and only 36% had bilateral groin dissection. Radical vulvectomy was reserved for patients with multifocal disease, while bilateral groin dissection was reserved for midline lesions, or those within 1−2 cm of an imaginary line drawn from the clitoris to the anus. Despite this more conservative surgical approach, the five-year DSS for all 345 patients was 86% and the five-year DSS for the 102 patients with positive lymph nodes was 66%.

Seventy-eight of the 111 recurrences (70.3%) were on the vulva, 69 of which were isolated vulvar recurrences. With further surgical excision and/or radiation therapy, 94% of patients with an isolated vulvar recurrence, including the 31 patients in this group who had more than one vulvar recurrence, were free of disease at five years. By contrast, of the 42 patients who had a groin, vaginal or distant recurrence, only 8% were alive at five years.

Although this study confirms the benefit of a more conservative approach to vulvar resection, the actual extent of the surgical margin has been controversial. Several recent studies have reported that the histopathological margin distance was not predictive of vulvar recurrence [12,13,14,18,22,23,24,25]. However, there have been two recent exceptions, A meta-analysis of 10 studies reported by Nooij et al. showed that a tumour-free margin of <8 mm was associated with a higher risk of local recurrence compared to a tumour-free margin of ≥ 8 mm (HR, 1.99; 95% CI: 1.13−3.51) [15], but in a cohort study of their own patients, they were unable to confirm this finding. Similarly, Yang et al., in a multicentre study of 335 patients, also reported that patients with surgical margins < 8 mm had a higher rate of local recurrences [26].

None of these studies was as large as the present study, and none subdivided the recurrences into those at the primary or at a remote site. In our univariable analysis, margin distance was not predictive of a vulvar recurrence per se, but when subdivided into primary and remote site recurrences, there was a significantly increased risk of primary site recurrence in patients with margins < 8 mm. In multivariable analysis, patients with a pathological margin <5 mm had a significantly increased risk of all vulvar and primary site recurrences, while those with a margin of 5 to <8 mm had a significantly higher risk of a primary site recurrence and lower risk of a remote site recurrence.

In a review of the literature, te Grootenhuis et al. concluded that the division into primary versus remote site recurrences was arbitrary and not reproducible [17]. Our results demonstrate that with large enough numbers, this division is reproducible if accurate clinical records of tumour locations have been kept. We agree that distinguishing a “true” local recurrence from a second primary cancer would require molecular profiling of both cancers, and two of our primary site recurrences occurred more than five years post-treatment. These were almost certainly second primary cancers. However, the division is important because it allows clear guidelines to be given regarding primary surgical treatment.

Due to the confusion in the literature regarding the significance of surgical margins, there has also been confusion regarding surgical recommendations. Woelber et al. recommended that the main goal should be to achieve “complete tumour resection, irrespective of tumour-free margin” [22], and te Grootenhuis et al. concluded from their systematic review that there seemed to be “no lower limit (apart from involved margins) below which further treatment to the vulva should be recommended” [17]. Nooij et al. and Preti et al. also suggested that “tumour positive margins” were the only risk factor [15,27]. Groenen et al. recommended removing “no more than sufficient surrounding tissue” [12], while others have recommended that margins should be at least 2 mm [23], 5 mm [28,29] or ≥8 mm [30]. 

In 1953, Slaughter et al. undertook a histopathological study of 783 squamous oropharyngeal cancers to better understand their natural history [31]. They concluded that these carcinomas arose from multiple areas which had been preconditioned by some carcinogenic agent, rather than from a single cell. They coined the term “field cancerization” and believed that such a concept would in part explain the high local recurrence rate of oral cancers. Over 50 years later, and with current knowledge of the molecular basis of cancer, Dakubo et al. explored the clinical implications of this concept in multiple cancers, including those of the head and neck, lung and vulva [32]. They concluded that there were two types of local recurrence—those that occurred at the primary site and those that occurred at a distant site. They called recurrences that were genetically similar to the primary “second field tumours” and those that were genetically dissimilar “second primary tumours”.

In the future, genetic sequencing of the primary tumour and histologically normal, but genetically transformed, surgical margins should be able to better identify patients at risk for primary site recurrences. Treatment of such patients by re-excision or radiation therapy should decrease the incidence of these recurrences and allow closer surgical margins in selected cases. Another possible approach in the future may be the use of electrochemotherapy [33]. However, until a reliable alternative becomes routine, we believe that surgical margins should ideally be 1 cm of macroscopic skin, which translates to a histopathological margin of 8 mm. This is consistent with current guidelines from the National Comprehensive Cancer Network (NCCN) [34] and the European Society of Gynaecological Oncology (ESGO) [35], which suggest that the surgical margin should be at least 1 cm.

Unlike several studies that questioned the benefit of treating close margins [12,13,18,24], we found treatment with radiotherapy or vulvar re-excision for margins < 8 mm to be associated with a lower rate of all vulvar and primary site recurrences in multivariable analysis. The beneficial effect of treatment was most apparent in those patients with margins < 5 mm. 

Based on these findings, we believe that patients whose surgical margins are <5 mm should undergo surgical re-excision if feasible. Treatment to prevent recurrence merely requires re-excision of the scar, whereas observation until a recurrence occurs will inevitably mean a wider excision, even if the recurrence is diagnosed early. If proximity to the clitoris, anus or distal urethra makes surgical resection inappropriate, or if radiation is required because of positive lymph nodes, a local field of radiation should be given. Patients whose margins are 5–7.9 mm could be followed closely if surgical re-excision were not appropriate. Follow-up should be for life and should include teaching the patient techniques for self-examination. The risk of recurrence in this group of patients, 22.2%, is not different from the risk of recurrence with margins ≥ 8 mm (23.3%), although over 90% of recurrences will be at the primary site in patients with close margins, while for patients with margins ≥ 8 mm, 86% of recurrences will be at a remote site.

In addition to having close or positive surgical margins, primary site recurrences occurred earlier than remote site recurrences, 66.7% occurring within two years compared to 31% of remote site recurrences. They were also significantly associated with positive groin nodes in multivariable analysis. 

In multivariable analysis, differentiated VIN at the margin was significantly associated with a primary site recurrence, as has been previously reported for local recurrence [18,26]. Others have found LS to be associated with an increased risk of local recurrence in multivariable analysis [16,25]. We could not confirm the latter finding.

An unexpected finding in this study was that current smokers had a lower rate of all vulvar recurrences than non-smokers, with a hazard ratio on multivariable analysis of 0.42. The level of evidence for this association was weaker when primary and remote site recurrences were examined individually. Yap et al. reported smoking to be protective against remote, but not primary site recurrences [16]. This may be related to the fact that smokers are generally younger and have more uVIN, and the latter was associated with a lower rate of recurrences in univariable analysis. Age over 65 years was also associated with a significantly lower rate of all vulvar recurrences on multivariable analysis, (HR 0.58), possibly related to the fact that local recurrences often occur many years post-treatment, and these elderly patients die of other causes first.

### Strengths and Limitations

To our knowledge, this study is the largest monocentric series in the literature focusing primarily on margin distance and site of local recurrence. All patients were treated in a high-volume tertiary referral centre and 70% of the patients were treated by the one surgeon (NFH). Surgical management and indications for adjuvant radiation were consistent throughout. Other strengths include the long duration of follow up (median 93 months), with all patients being followed at least annually for life. The main limitation is that it is a retrospective study, but sites of primary and recurrent disease were carefully recorded prospectively. The histopathology was not retrospectively reviewed. All slides were reported by a specialist gynaecological pathologist, although there were several different pathologists involved over the 29-year period. 

## 4. Materials and Methods

### 4.1. Study Design

**Figure 5:**
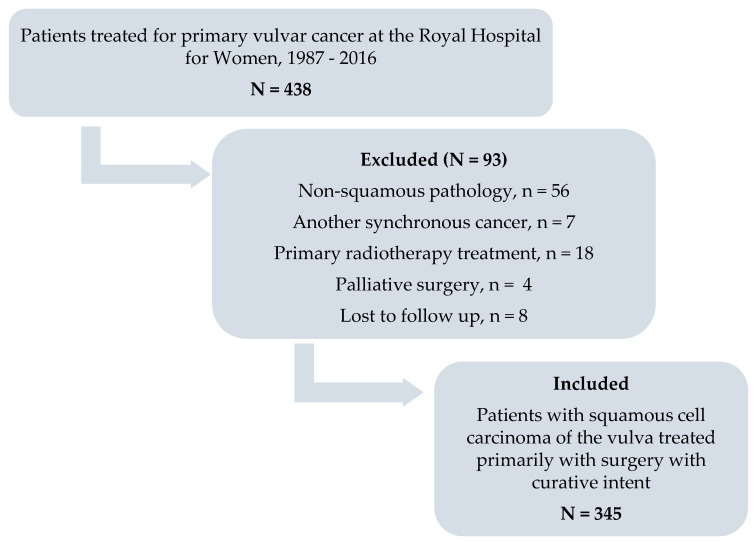
Flow diagram to illustrate patients included and excluded from the study.

This study was a retrospective review of patients with squamous cell carcinoma of the vulva, treated primarily with surgery with curative intent, at the Royal Hospital for Women, Sydney. The study was conducted in accordance with the Declaration of Helsinki, and the protocol was approved by the South Eastern Sydney Local Health District Human Research Ethics Committee (Reference number: 15/151(LNR/POWH/311). The departmental database was reviewed for consecutive patients treated for primary carcinoma of the vulva between February 1987 and December 2016 (*n* = 438). Figure 5 describes the inclusion and exclusion criteria for the study group. Demographic, clinical, surgical, histopathological, 2009 FIGO staging and outcome data were extracted from the hospital’s medical records. Patients were followed until death from any cause, or until the end of data extraction on 31 July 2019. 

### 4.2. Histopathological Margins and Recurrence

The closest histopathological invasive cancer-free skin margin measured in millimetres on haematoxylin and eosin-stained slides was retrospectively retrieved from the histopathology report. The margin was measured by a specialist gynaecological pathologist from the peripheral margin of the invasive cancer to the inked skin margin of the specimen. Positive margins were defined as invasive carcinoma at any surgical skin edge. The presence of any associated lichen sclerosus in the specimen was also retrieved from the pathology report, as was the presence of any vulvar intraepithelial neoplasia (VIN). Different terminologies were used for VIN over the course of the study, but we classified all lesions as either usual VIN, or differentiated VIN. For this study, a vulvar recurrence was defined as any invasive recurrence located on the vulva. Vulvar recurrences were subdivided into primary site recurrences (when they recurred within 2 cm of the primary resection scar) and remote site recurrences (when they recurred more than 2 cm from the primary scar). 

### 4.3. Surgical Treatment

Over the study period, we performed radical local excision or modified radical vulvectomy [6], initially aiming for a skin margin of 2−3 cm, with the deep margin being the fascia overlying the urogenital diaphragm. After the publication of the paper by Heaps et al. [9] in 1990, we aimed for a skin margin of 1 cm. This margin was drawn with a marking pen before stretching the skin for excision. Any clinically apparent usual or differentiated VIN was superficially resected with 5 mm margins, but no attempt was made to excise lichen sclerosus. If the cancer was within 5 mm of the clitoris or anus, primary radiation was used, and those patients were excluded from this study. If it were adjacent to, or encroaching on, the distal urethra, up to 1.5 cm of urethra was resected if that would provide a 1 cm margin. All patients with stages IB and above had some type of groin node evaluation. Most had at least a unilateral inguino-femoral lymphadectomy, but if there were palpably enlarged nodes, these were resected and sent for frozen section. If positive, complete groin dissection was not performed, and post-operative groin and pelvic radiation was given [36,37]. In recent times, some patients underwent sentinel node biopsy, or ultrasonic groin surveillance for early lesions, on a research protocol, if the tumours were 15 mm or less in diameter with a depth of invasion of 3 mm or less. 

### 4.4. Radiotherapy Treatment

Sixty-eight patients received some form of post-operative radiation. Of these, 54 (79%) were treated at the adjacent Prince of Wales Hospital and 14 (21%) were treated at various other city or regional cancer centres, usually in consultation with a radiation oncologist from our team. Of the 68 patients receiving radiation, 22 patients received radiation to the groins and pelvis for positive nodes, and 39 received radiation to the vulva, groins and pelvis for close surgical margins and positive nodes (*n* = 31), or multifocal disease and positive nodes (*n* = 8). Seven patients received radiation to the vulva only. Of these, five had close or positive surgical margins and negative or 1−2 microscopically positive groin nodes, one had dVIN at the margin, and another had multifocal disease, a positive margin for uVIN and immunosuppression following an organ transplant. A small, direct electron beam field was used, based on a margin of 2−3 cm around the scar. The dose ranged from 54 to 60 Gy, with up to 65 Gy given for gross residual disease. Patients with multifocal vulvar disease received a similar dosage via a direct electron field to the whole vulva. 

Various techniques and dosages were used over the 29-year period to treat the groins and pelvis. Since 2010, intensity modulated radiotherapy (IMRT) or volumetric modulated arc therapy (VMAT) were used to give a more uniform dose to the vulva (if incorporated into the field), groins or lower pelvic lymph nodes as required. Bolus was used to increase skin dose if needed. If the vulva required a higher dose than the nodes, this was incorporated into the VMAT plan or given as a separate electron field at the end of treatment. Patients were treated with legs together for comfort or slightly apart (frog leg position) to reduce unnecessary dose to the inside of the thigh. Concurrent chemotherapy was not normally used in this older population. 

Any lymph nodes greater than 1.5 cm were resected unless fixed, so treatment for positive nodes (*n* = 61) usually involved a dose of 45–50 Gy to the nodal bed. Patients were planned with a computed tomographic (CT) scan, using intravenous contrast to outline the vessels. Patients with extracapsular nodal spread were boosted to a dose of 54 Gy, and higher for any macroscopic residual disease. 

### 4.5. Statistical Analysis

All analyses were performed using IBM SPSS Statistics for Windows (version 26) and R (version 4.0) [38,39]. Descriptive statistics were used to summarize patient demographics and clinicopathological variables and are presented as frequencies or medians. Median follow-up was calculated using the reverse Kaplan–Meier method [40]. 

For the primary analysis, the outcome of interest, time to first local recurrence, was calculated from the date of surgery until the date of disease recurrence on the vulva, or date of last follow-up. The local recurrence rate was calculated using the Kaplan–Meier method, and associations between various margin groups and treatment factors with local recurrence were assessed using the log rank test. A *p* value of 0.05 or less was considered statistically significant.

Cox proportional hazard models [41] were used in univariable and multivariable analyses to estimate the associations between all vulvar recurrences, and potential clinicopathological risk factors. Three margin sub-groups were used in these models: <5mm (including positive margins), 5 mm to <8 mm, and ≥8 mm, but only a sub-group of <8 mm was used to determine association with treatment. To examine the competing risks of primary and remote site vulvar recurrence, we fit univariable and multivariable proportional subdistribution hazards models [42] to each outcome, using the same covariates as for the main analysis. Hazard ratios (HR), or subdistribution hazard ratios (SHRs) as appropriate and the corresponding 95% confidence intervals (CI) are presented. 

A secondary analysis, using the Kaplan–Meier method, was conducted to compare five and ten-year progression-free survival (PFS) and disease specific survival (DSS) with 2009 FIGO stage for the entire study group. Five and ten-year DSS was also calculated to determine potential survival differences between patients with an isolated vulvar recurrence and with a regional or distant recurrence. Five and ten-year DSS and overall survival (OS) were estimated for the whole study cohort. PFS was defined from the date of surgery until the date of disease recurrence or last follow-up. DSS was defined from date of surgery to the date of death due to vulvar cancer. All other patients were censored at date of last follow-up, or date of death from another cause, without a vulvar cancer recurrence. OS was determined from date of surgery to the date of death from any cause, or last follow-up. The two-sided log-rank test was used to calculate survival comparisons.

## 5. Conclusions

In summary, our study has demonstrated that conservative vulvar resection (radical local excision) in 80% and bilateral inguino-femoral lymphadenectomy in 35.6% of patients with squamous cell carcinoma of the vulva is associated with an excellent survival. Although surgical margins < 8 mm were not associated with an increased risk of all vulvar recurrences in univariable analysis, when broken down into primary and remote site recurrences, margins < 8 mm were significantly associated with an increased risk of primary site vulvar recurrence. In multivariable analysis, primary site recurrences were significantly increased in patients with margins < 8 mm, and all vulvar and primary site recurrences in patients with margins < 5 mm. Primary site recurrences were also significantly increased in patients with one or more positive nodes, or with differentiated VIN at the excision margin. Patients with close or positive margins who were treated with either surgical re-excision or radiotherapy had a significantly decreased risk of recurrence. Our results support the recommendation that excision margins should be at least 1 cm, which equates to a histopathological margin of 8 mm. Surgical re-excision or radiation therapy should be recommended if the histopathological margin is <5 mm. Future genetic analysis of the skin adjacent to the cancer may allow closer margins in selected cases.

## Figures and Tables

**Table 1:** Clinical, surgical, and pathological characteristics of the vulvar. Squamous cell carcinoma cohort (*n* = 345).

Variable	N (%)
Median follow-up	93 months (range 1–367)
Age, years (range 29–96)	
Mean	66.7
Median	70
FIGO Stage 2009	
IA	37 (10.7%)
IB	194 (56.2%)
II	12 (3.5%)
IIIA ^(1)^	35 (10.1%)
IIIA ^(11)^	14 (4.1%)
IIIB ^(1)^	5 (1.5%)
IIIB ^(11)^	11 (3.2%)
IIIC	32 (9.3%)
IVA ^(11)^	2 (0.6%)
IVB	3 (0.9%)
Smoking	
Current	91 (26.4%)
Past	53 (15.3%)
Never	201 (58.3%)
Groin node status	
Unknown	1 (0.3%)
Negative	242 (70.1%)
Positive	102 (29.6%)
Lesion Location	
Clitoris	39 (11.3%)
Labium minus	95 (27.5%)
Labium majus	118 (34.2%)
Perineum	15 (4.3%)
Vulvar vestibule	35 (10.1%)
Multifocal	43 (12.5%)
Primary surgery	
Radical local excision	275 (79.7%)
Radical vulvectomy	70 (20.3%)
Primary groin treatment	
Bilateral IGFLND	123 (35.6%)
Unilateral IGFLND ^a^	122
Nodal debulking ^a^	47
Sentinel node ^a^	5
No groin node dissection	63 (18.3%)
Adjuvant radiotherapy	
vulva/groins/pelvis ^b^	39 (11%)
groins/pelvis	22 (6.1%)
vulva	7 (1.2%)
Epithelial disorder	
uVIN	115 (33.3%)
dVIN	77 (22.3%)
Lichen sclerosus (LS)	77 (22.3%)
LS + dVIN	26 (7.5%)
uVIN + dVIN	1 (0.3%)
None	49 (14.2%)
Margin distance ^c^	
Positive	11 (3.2%)
0.1 mm – 7.9 mm	111 (32.5%)
≥8 mm	219 (64.2%)
Tumour Size	
≤4 cm	286 (82.9%)
>4 cm	59 (17.1%)
LVSI	
Yes	53 (15.4%)
No	291 (84.3%)
Unknown	1 (0.3%)
Perineural invasion	
Yes	24 (6.9%)
No	318 (92.2%)
Unknown	3 (0.9%)
Depth of invasion	
≤5 mm	222 (64.3%)
>5 mm	123 (35.7%)
Differentiation	
Well	153 (44.3%)
Moderate	152 (44.1%)
Poor	39 (11.3%)
Unknown	1 (0.3%)

FIGO, International Federation of Gynecology and Oncology; IGFLND, inguino-femoral lymph node dissection; uVIN, usual-type vulvar intraepithelial neoplasia; dVIN, differentiated vulvar intraepithelial neoplasia; LVSI, lymphovascular space invasion. ^a^ Different groin procedure in alternate groins are included in each group (% not calculated). ^b^ Four patients had chemoradiation, ^c^ four patients excluded due to follow-up < 6 months.

**Table 2:** Univariable Cox and Fine-Gray regression analyses comparing all vulvar recurrences, and primary and remote site vulvar recurrences with clinico-pathological variables.

Variable Title	All Vulvar Recurrence	Primary Vulvar Recurrence	Remote Vulvar Recurrence
	HR (95% CI) *p*-value	SHR (95% CI) *p*-value	SHR (95% CI) *p*-value
Age > 65 years (Ref: ≤65 years)	1.16 (0.74−1.82) 0.52	1.38 (0.69−2.77) 0.36	0.98 (0.54−1.77) 0.94
Current smoker—Yes (Ref: No)	0.33 (0.17−0.64) 0.001	0.34 (0.12−0.96) 0.04	0.37 (0.15−0.86) 0.02
Tumour size >4 cm (Ref: ≤4 cm)	1.22 (0.64−2.32) 0.54	1.79 (0.77−4.15) 0.17	0.69 (0.24−1.98) 0.49
Margin distance (Ref: ≥8 mm)			
<5mm margins	1.17 (0.64−2.16) 0.61	8.82 (3.54−21.99) < 0.001	0.00 ^†^
5 mm to < 8 mm margins	1.06 (0.59−1.92) 0.84	7.59 (3.04−18.95) < 0.001	0.07 (0.01−0.54) 0.01
Margin treatment			
< 8mm treated margins (Ref: all other margins)	0.34 (0.12−0.93) 0.04	0.95 (0.34−2.70) 0.93	0.00 ^†^
Nodal status ^a^—Positive (Ref: negative)	1.82 (1.15−2.89) 0.01	3.77 (1.91−7.46) < 0.001	0.81 (0.40−1.65) 0.57
Tumour differentiation			
Mod/Poor (Ref: well differentiated)	1.29 (0.82−2.03) 0.27	1.92 (0.92−4.03) 0.08	0.93 (0.52−1.66) 0.81
Lymphovascular invasion—Yes (Ref: No)	1.67 (0.90−3.09) 0.10	2.67 (1.21−5.91) 0.02	0.79 (0.27−2.30) 0.67
Perineural invasion—Yes (Ref: No)	1.95 (0.78−4.86) 0.15	3.12 (1.08−9.06) 0.04	0.56 (0.07−4.33) 0.58
Depth of invasion—>5 mm (Ref: ≤5 mm)	1.23 (0.77−1.97) 0.39	1.51 (0.76−2.99) 0.24	0.97 (0.51−1.82) 0.92
Epithelial abnormality (Ref: No abnormality) ^b^			
Lichen sclerosus (LS) +/- SH	1.63 (0.80−3.29) 0.18	2.40 (0.66−8.77) 0.19	1.20 (0.51−2.83) 0.68
dVIN present, not at margin	0.98 (0.44−2.19) 0.96	2.18 (0.57−8.35) 0.25	0.50 (0.17−1.51) 0.22
dVIN at margin	2.07 (0.88−4.89) 0.10	3.73 (0.87−15.95) 0.08	1.21 (0.41−3.60) 0.73
uVIN present	0.43 (0.20−0.96) 0.04	0.60 (0.14−2.55) 0.49	0.40 (0.15−1.05) 0.06
LS + dVIN	0.78 (0.25−2.47) 0.68	0.73 (0.08−6.81) 0.78	0.84 (0.24−2.95) 0.79

HR indicates hazard ratio; SHR, subdistribution hazard ratio; CI, confidence interval; Ref, Reference group; LS, lichen sclerosus; SH, squamous hyperplasia; dVIN, differentiated vulvar intraepithelial neoplasia; uVIN, usual-type vulvar intraepithelial neoplasia; ^a^ 1 patient nodal status unknown, ^b^ 1 uVIN + dVIN excluded. ^†^ The SHR could not be estimated as no remote recurrences were observed in the <5 mm and treated margin groups.

**Table 3:** Multivariable Cox and Fine–Gray regression analyses comparing all vulvar recurrences, and primary and remote vulvar recurrences to clinico-pathological variables.

Variable Title	All Vulvar Recurrence	Primary Vulvar Recurrence	Remote Vulvar Recurrence
	HR (95% CI) *p*-value	SHR (95% CI) *p*-value	SHR (95% CI) *p*-value
Age >65 years (Ref: ≤65 years)	0.58 (0.33−1.00) 0.05	0.49 (0.18−1.33) 0.16	0.62 (0.31−1.23) 0.17
Current smoker—Yes (Ref: No)	0.42 (0.20−0.87) 0.02	0.51 (0.16−1.62) 0.26	0.41 (0.16−1.05) 0.06
Tumour >4 cm (Ref: ≤4 cm)	1.34 (0.64−2.82) 0.44	2.41 (0.57−10.13) 0.23	0.84 (0.24−2.94) 0.79
Margin distance (Ref: ≥8 mm)			
<5 mm margins	2.29 (1.12−4.70) 0.02	15.19 (5.21−44.26) < 0.001	0.00 ^†^
5 mm to <8 mm margins	1.31 (0.69−2.49) 0.41	8.92 (3.26−24.43) < 0.001	0.08 (0.01−0.71) 0.02
Margin treatment			
<8 mm treated margins (Ref: all other margins)	0.15 (0.05−0.44) < 0.001	0.14 (0.05−0.41) < 0.001	0.00 ^†^
Nodal status ^a^—Positive (Ref: negative)	1.77 (1.05−3.01) 0.03	3.05 (1.18−7.89) 0.02	1.07 (0.49−2.32) 0.86
Tumour differentiation			
Mod/Poor (Ref: well differentiated)	1.17 (0.70−1.96) 0.54	1.28 (0.50−3.27) 0.61	1.25 (0.65−2.40) 0.50
LVSI present (Ref: not present)	1.62 (0.77−3.42) 0.21	1.07 (0.33−3.47) 0.91	2.74 (0.73−10.29) 0.14
PNI present (Ref: not present)	1.72 (0.61−4.88) 0.31	2.06 (0.51−8.30) 0.31	0.50 (0.03−7.11) 0.61
Depth of invasion—>5 mm (Ref: ≤5 mm)	0.77 (0.44−1.36) 0.37	0.66 (0.26−1.68) 0.38	0.96 (0.47−1.95) 0.91
Epithelial abnormality (Ref: No abnormality) ^b^			
Lichen sclerosus (LS) +/- SH	1.72 (0.81−3.65) 0.16	2.34 (0.55−9.92) 0.25	1.46 (0.60−3.53) 0.40
dVIN present, not at margin	0.96 (0.41−2.27) 0.93	1.59 (0.40−6.31) 0.51	0.70 (0.19−2.60) 0.59
dVIN at margin	2.37 (0.94−5.99) 0.07	5.35 (1.05−27.34) 0.04	1.65 (0.56−4.87) 0.36
uVIN present	0.42 (0.17−1.02) 0.06	0.47 (0.09−2.45) 0.37	0.46 (0.17−1.27) 0.13
LS + dVIN	0.59 (0.18−1.97) 0.39	0.52 (0.05−5.58) 0.59	0.96 (0.24−3.90) 0.96

HR indicates hazard ratio; SHR, subdistribution hazard ratio; CI, confidence interval; Ref, Reference group; LVSI, lymphovascular space invasion; PNI, perineural invasion; SH, squamous hyperplasia; dVIN, differentiated vulvar intraepithelial neoplasia; uVIN, usual-type vulvar intraepithelial neoplasia. ^a^ One patient nodal status unknown, ^b^ One patient with both dVIN and uVIN excluded from analysis. ^†^ The SHR could not be estimated as no remote recurrences were observed in the <5 mm and treated margin groups.

## Supplementary Materials

The following are available online at https://www.mdpi.com/2072-6694/12/11/3375/s1, Figure S1. Kaplan–Meier curve for ten-year disease specific survival for the whole study cohort; Figure S2. Kaplan–Meier curve for ten-year disease-specific survival for patients with positive nodes; Figure S3. Kaplan–Meier curve for ten-year disease-specific survival stratified by site of first recurrence; Figure S4. Kaplan–Meier curve for ten-year overall survival for the total study cohort; Figure S5. Local recurrence rate in margins ≥ 8 mm compared to margins < 8 mm; Table S1. margin distance and treatment of positive or close margins to site of vulvar recurrence; Table S2. Breakdown of vulvar recurrence per mm margin distance in 25 patients with untreated margins < 5 mm.
