# Supplementary material for: The Prognostic Role of the Surgical Margins in Squamous Vulvar Cancer: A Retrospective Australian Study
Source: Cancers (Basel). 2020 Nov 14;12(11):3375. doi: 10.3390/cancers12113375 (PMC7697402; doi:10.3390/cancers12113375)
Supplement: Supplementary file 1 [file cancers-12-03375-s001.pdf]

cancers

MDPI

Article

Supplementary Material: The Prognostic Role of the Surgical Margins in Squamous Vulvar Cancer: a Retrospective Australian Study

Ellen L Barlow [1]*, Michael Jackson [2,3] and Neville F Hacker [1,4]

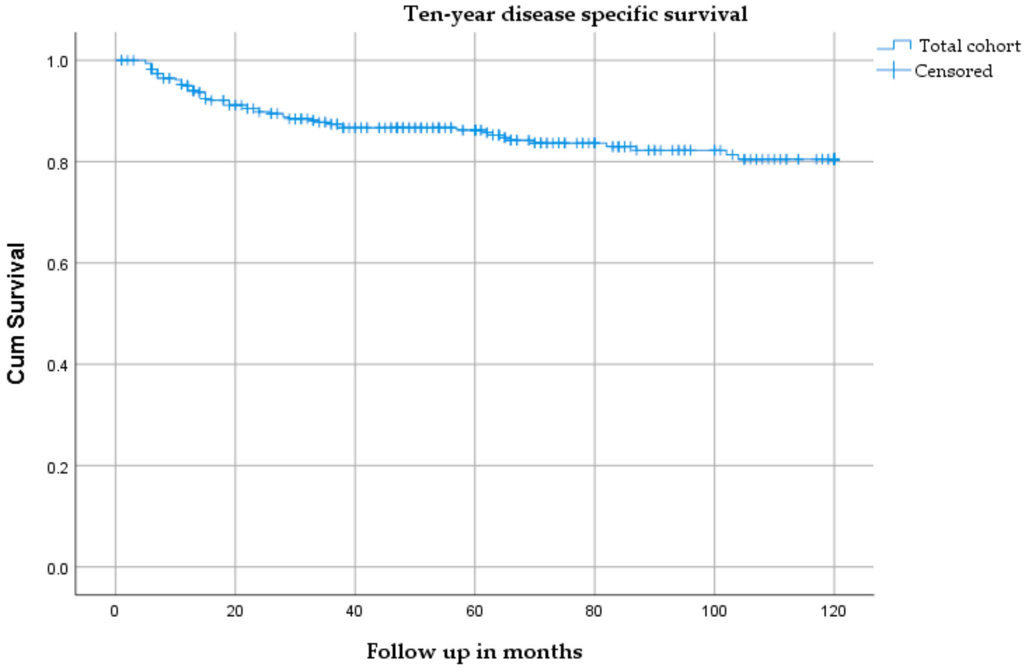

Figure S1. Kaplan-Meier curve for ten-year disease specific survival for the whole study cohort (n = 345).

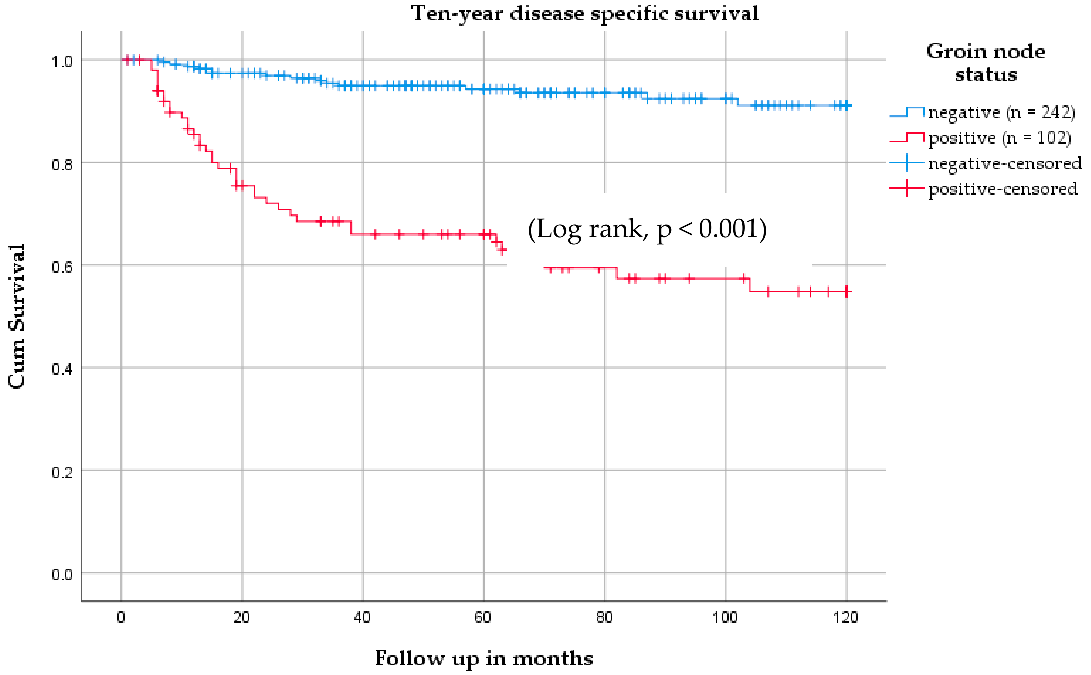

Figure S2. Kaplan-Meier curve for ten-year disease-specific survival stratified by nodal status.

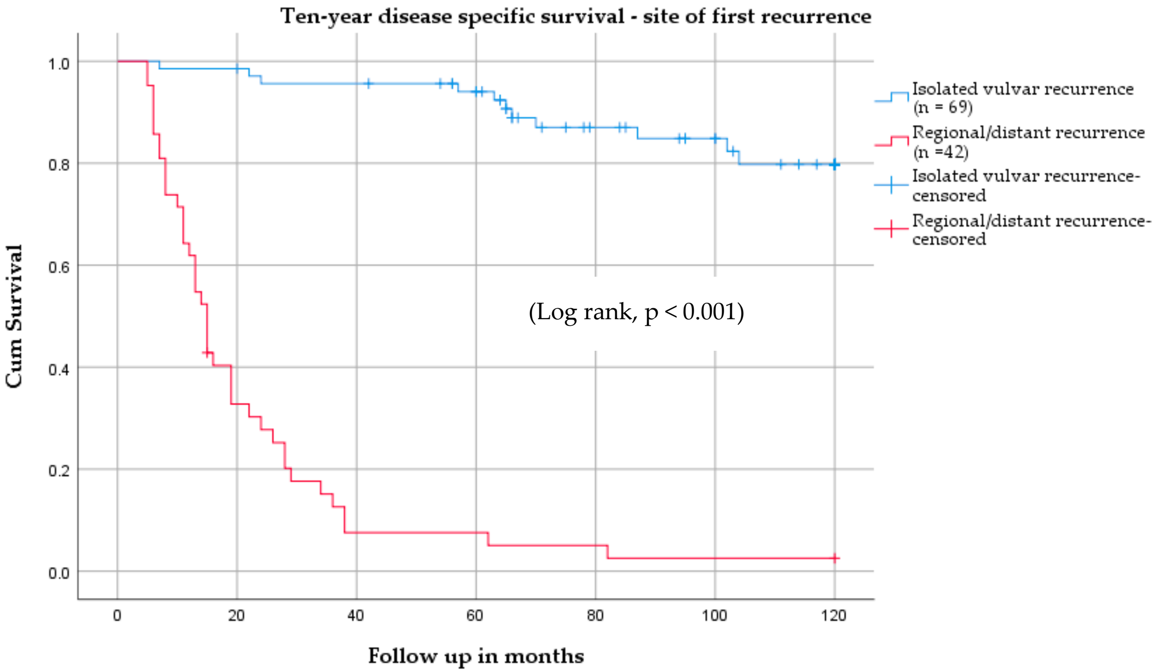

Figure S3. Kaplan-Meier curve for ten-year disease specific survival stratified by site of first recurrence.

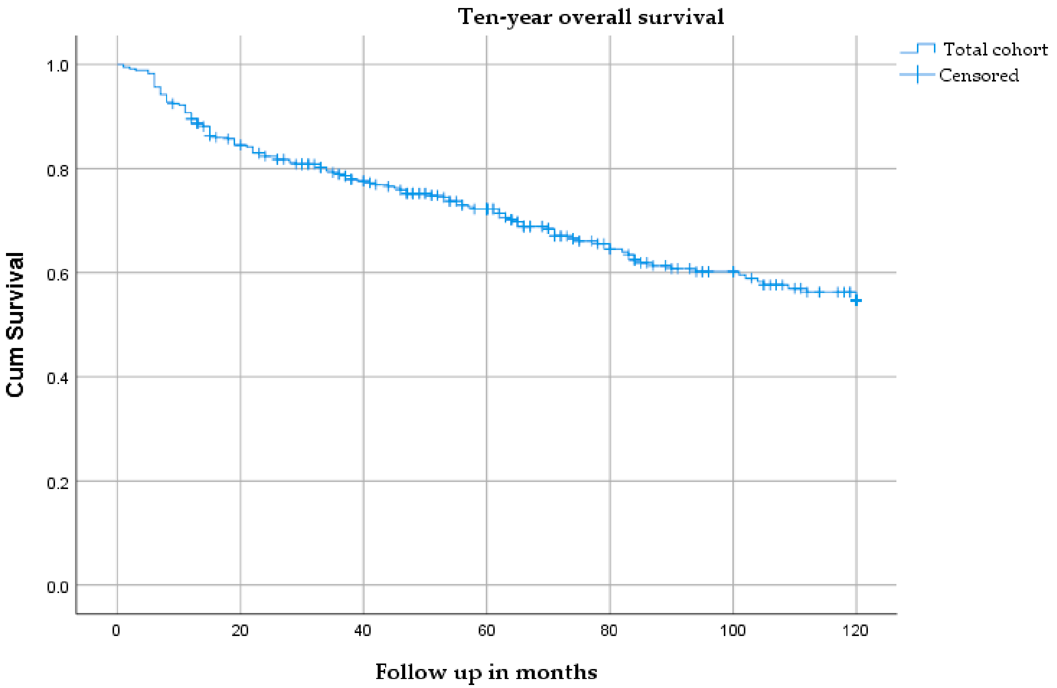

Figure S4. Kaplan-Meier curve for ten-year overall survival for the total study cohort (n = 345).

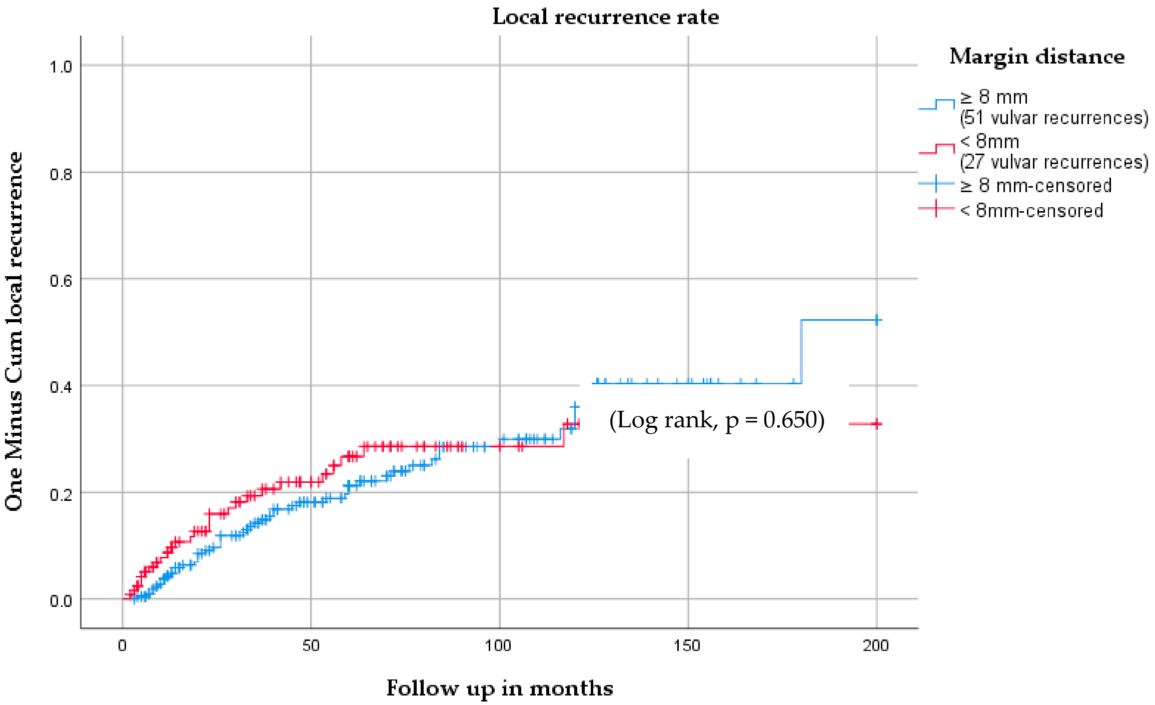

Months	0	20	40	60	80	100	120	180
< 8 mm margin	122	85	62	44	27	20	15	3
≥ 8 mm margin	219	172	131	102	68	49	33	5

Number at risk

Figure S5. Local recurrence rate in 219 patients with margins ≥ 8 mm and in 122 patients with margins < 8 mm.

Table S1. Margin distance and treatment of positive or close margins to incidence and site of vulvar recurrence.

Margin distance	Total n	Vulvar recurrence n (%)	P value[a]	Primary site recurrence (n =33)	Remote site[b] recurrence (n = 45)
*Positive margin	11	2 (18.2%)			
- treated with radiotherapy	8	2 (25%)		2	0
- treated with re-excision	3	0 (0%)	0.157		
Close margins					
0.1 mm – 4.9 mm treated margins	23	1 (4.3%)			
0.1 mm – 4.9 mm untreated margins	25	10 (40%)	0.003		
- treated with radiotherapy	14	0 (0%)			
- untreated margins	25	10 (40%)	0.009	11	0
- treated with re-excision	9	1 (11.1%)			
- untreated margins	25	10 (40%)	0.095		
5 mm – 7.9 mm treated margins	12	1 (8.3%)			
5 mm – 7.9 mm untreated margins	51	13 (25.5%)	0.165		
- treated with radiotherapy	9	1 (11.1%)			
- untreated margins	51	13 (25.5%)	0.352		
- treated with re-excision	3	0 (0%)		13	1
- untreated margins	51	13 (25.5%)	0.252		
< 8 mm treated margins	76	23 (30.2%)			
< 8 mm untreated margins	46	4 (8.7%)	0.005		
Wide margins					
≥ 8 mm margins	219	51 (23.3%)		7	44

*All positive margins treated with either radiotherapy or re-excision. [a] Log-rank test. [b] no remote site recurrences in positive, or 0.1 - 4.9 mm margins.

Table S2. Breakdown of vulvar recurrence per mm margin distance in 25 patients with untreated margins < 5 mm.

Margin distance	Number	Local recurrence
1 mm	2	0 (0%)
2 mm	4	1 (4%)
3 mm	5	3 (12%)
4 mm	14	6 (24%)
Total	25	10 (40%)

© 2020 by the authors. Licensee MDPI, Basel, Switzerland. This article is an open access article distributed under the terms and conditions of the Creative Commons Attribution (CC BY) license (http://creativecommons.org/licenses/by/4.0/).
